# Exploring a metacognitive approach for case analysis based learning of anxiety adjustment in nurses: a qualitative study

**DOI:** 10.5116/ijme.66af.82fc

**Published:** 2024-08-23

**Authors:** Yuji Iwama, Mitsuru Ikeda

**Affiliations:** 1 Department of Nursing, National Defense and Medical College, Tokorozawa, Japan; 2 School of Knowledge Science, Japan Advanced Institute of Science and Technology, Nomi, Japan

**Keywords:** Anxiety, metacognitive approach, scaffold, nurse, qualitative study

## Abstract

**Objectives:**

This study aimed to investigate the effectiveness of a metacognitive learning program in enhancing nurses’ anxiety adjustment skills.

**Methods:**

A qualitative exploratory study was conducted using semi-structured individual interviews with Japanese nurses who participated in a metacognitive learning program at Keio University Hospital. The program included a 90-minute classroom workshop followed by five online reflection sessions over three months. Participants were selected through purposive sampling, targeting those with at least four years of oncology nursing experience. Of the nine program participants, five met the inclusion criteria and consented to participate. Data were recorded and interviews transcribed three months post-program completion and analyzed using “Steps for Coding and Theorization.”

**Results:**

Two main themes emerged from the analysis. The first theme, “Development of Metacognitive Understanding,” highlighted the participants' progress in comprehending metacognition through structured exercises and reflection. The second theme, “Cultivating Anxiety Adjustment Skills through Metacognition,” revealed improvements in coping with anxiety, including increased selfawareness, proactive strategies, and the application of metacognitive techniques in clinical practice.

**Conclusions:**

This study found that metacognitive approaches could promote anxiety adjustment skills among nurses. Using learning scaffolds and reflecting on personal experiences, nurses could strengthen their metacognitive skills to adjust anxiety. These findings suggest that incorporating metacognitive approaches in nursing education could be beneficial. Future research should explore this approach in diverse clinical settings to generalize the results and examine the long-term impacts on nurses' anxiety management.

## Introduction

As a profession, nursing is fraught with difficulties, and often accompanied by significant anxiety.^1^^-^^5^Anxiety tends to occur when nurses perceive a lack of skills to meet the increasing care demands of patients, or when they face unresolved conflicts.^6^^,^^7^ This is particularly prevalent in scenarios involving cancer patients, where empathetic care and emotional support are central to nursing roles.^8^ Nurses’ mental well-being and efficient performance are significantly impacted by this stress.^6^^,^^9^ Challenges in communicating with patients can amplify stress levels and potentially lead to an increased risk of burnout syndrome.^10^ Anxiety can also affect the quality of patient care provided by nurses, as well as their ongoing learning. It has been stated that anxiety can lead nurses to adopt passive coping mechanisms, or avoidance behaviors.^8^^,^^9^ Such passive attitudes prevent nurses from fulfilling patients’ high support needs, thereby leading to decreased satisfaction. Avoidance also prevents nurses from gaining experience, which could lead to an increase in anxiety-inducing situations. This vicious cycle is a significant issue for nurses, especially in Japan, where the incidence rate of cancer exceeds one million annual cases.^11^ This makes the management of nurses’ anxiety a major social challenge.^12^^-^^14^

Resilience is said to be the key to coping with anxiety.^8^^,^^15^^,^^16^ Resilience can be divided into two main components: 1)the ability to suppress discomfort, which can lead to job dissatisfaction or burnout when feeling anxious,^6^ and 2) the cognitive ability to organize situations and make patient care-related decisions when anxious.^16^ The effectiveness of mindfulness-centered educational programs in the former have been widely reported.^17^^-^^19^Marconi and colleagues who conducted an 18-week mindfulness program for healthcare workers, reported that participants showed improved coping strategies and reduced anxiety levels after experiencing stress.^18^ Conversely, nurses face the necessity of meeting patients’ needs while confronting their anxiety in the medical field. Therefore, developing cognitive skills to control one’s emotions are essential. However, cognitive education focusing on anxiety adjustment has not been well studied.

In this study, we focused on metacognition and developed a learning program specifically designed for anxiety adjustment in nurses. Metacognition, a concept introduced in the 1970s by Fravell as, “thinking about thinking,” encompasses understanding and managing one’s own cognitive processes.^20^ In the medical field, practicing metacognition involves monitoring one’s problem-solving thoughts and adjusting these thoughts and actions in appropriate directions, leading to improved problem-solving capabilities and increased confidence.^21^^-^^23^This approach has also been suggested to be effective in emotional adjustment,^15^^,^^24^ indicating its potential as a skill for nurses prone to anxiety, to mitigate its effects, and for continuous learning. However, current anxiety management programs for healthcare professionals lack focus on metacognitive skills and fail to address high-stress areas like oncology, creating a significant gap in effective anxiety coping strategies.

This study aimed to investigate the efficacy of metacognitive approaches in nurses’ learning of anxiety adjustment. Our findings are significant because they address this gap in cognitive education for anxiety adjustment among nursing professionals. By exploring the impact of metacognitive education on nurses’ ability to manage anxiety, this study provides valuable insights into effective anxiety adjustment strategies that are expected to improve nurses’ well-being and enhance patient care by providing nurses with the necessary tools to effectively manage their anxiety.

## Methods

### Study Design

This study utilized qualitative exploratory study to investigate the effectiveness of a metacognitive learning program in enhancing nurses' anxiety adjustment skills. Semi-structured individual interviews were chosen for their ability to capture subtle changes in metacognitive processes throughout the program.^25^

### Details of an anxiety adjustment learning program based on a metacognitive approach

The program consisted of a 90-minute classroom workshop, followed by five subsequent online reflection sessions, that were held immediately after the workshop, and then two weeks, one month, two months, and three months later, as outlined in Appendix A.Its primary learning objective was to empower nurses to adjust their anxiety by using metacognition, which is highly implicit and not usually recognized by nurses in clinical settings. Learning metacognition is difficult, because steps to reach this state have not been formulated. Therefore, to support learners in overcoming their learning barriers, the learning program provided them with metacognitive process descriptions developed from interviews with expert nurses about their anxiety adjustment experiences, as a learning scaffold. This educational approach centered on analyzing cases involving this metacognitive process.

The workshop consisted of three main sections: 1) a lecture on metacognition and anxiety, 2) a case analysis on adjusting anxiety using the learning scaffold of metacognitive thought, and 3) a reflection on one’s anxiety experiences using metacognition. The case of nurses’ anxiety adjustment was created through interviews with nurses in a preliminary study, in which, nurses felt anxious during dietary instructions in outpatient oncology care.^25^ Two cases were distributed to participants in paper form. In the first, the nurse used metacognition to provide care while adjusting for anxiety, and in the second, when metacognition did not work, the nurse had an avoidant response to patient care. The participants were asked to analyze the workings of metacognition for the two different cases, while using a metacognitive analysis model of nurses’ anxiety adjustment developed by the authors. Following their analysis, they reflected on and verbalized their anxiety experiences using the learning scaffold, and received feedback. During the workshop, we repeatedly explained to the participants that the learning scaffold provided to them was not the only correct answer, and that their learning goal was to be able to metacognitively self-introspect about their anxiety. After the workshop, participants reflected on their own clinical experiences with anxiety, using Google Form assessments five times over a three-month period to build their metacognition, and received feedback from the researcher after each session. To enable participants to metacognitively, reflect without relying on the scaffolding, during the online sessions, it was explained to them that they should reflect on their own, without scaffolding, as far as possible. Each workshop had one to three participants and used the same content in every session.

### Participants

Participants in this study were nurses with experience in oncology department, who participated in a learning program at Keio University Hospital between January and June 2021. The program was open to all nurses working in the hospital’s outpatient departments.

Recruitment initially required approval from the Director of Nursing and involved posting advertisements on departmental bulletin boards. These advertisements contained comprehensive information about the program, including its objectives, participant benefits, and its role in the study. The application period spanned from November 2019 to March 2021. Interview participants were required to have a minimum of four years of experience in oncology nursing in an outpatient department and completion of the learning program. These criteria were chosen to ensure participants had adequate practical experience and could provide valuable insight into program's impact on anxiety adjustment. Of the nine nurses who attended, five participated in the study (Appendix B), four were excluded for not meeting study criteria. Two declined interviews for personal reasons post-course completion, and two withdrew halfway through the course, rendering them ineligible for the post-program evaluation.

Before conducting interviews, participants received oral and written information about the study, including details on voluntary participation and data management. Participants chose a convenient interview location and we emphasized the confidentiality of data. This study obtained ethical approval from the Ethics Committee of the Japan Advanced Institute of Science and Technology and adhered strictly to guidelines to protect and maintain the confidentiality of participants' data.

### Data Collection

Participants initially completed the learning program. Semi-structured interviews were conducted between October 2021 and May 2022, approximately three months post-program completion. All interviews were conducted in Japanese by the first author in a private setting to ensure confidentiality. An interview guide was developed (Appendix C), and piloted with two participants. As no adjustments to the questions were necessary and the data collected were deemed significant, the pilot interviews were also included in the analysis. Interviews, lasting 30 to 50 minutes each, were recorded with the participants’ consent using a digital recorder.

This study employed purposive participant recruitment, targeting individuals who volunteered for the learning program. Despite diverse experiences, the focus on metacognitive learning facilitated theme development. Data saturation was achieved when no new information emerged from the interviews.^26^ In this study, we identified recurring patterns and themes among participants, confirming data saturation.

### Data Analysis

The transcripts were analyzed using Steps for Coding and Theorization (SCAT), a four-step coding process comprising: 1) identifying themes and constructs, 2) weavings themes and constructs together to develop a story, 3) writing a story-line, and 4) proposing theoretical descriptions based on the story-line.^27^^,^^28^ SCAT is characterized by its focus on seeking meaning in narratives, and applicability to small datasets. It has been used in medical education and nursing research and is known for its clarity and rigor in qualitative analyses.^29^^-^^32^

### Trustworthiness and Rigor

The first author (YI), a nurse with extensive experience within hospital settings, provided crucial contextual understanding for conducting and interpreting the interviews. The second author (MI), an expert in instructional design, specializing in methods that enhance metacognitive skills and transform learners' perceptions contributed complementary expertise essential for structuring the study and analyzing the data focusing on cognitive and perceptual shifts. YI conducted and transcribed all interviews. To ensure reliable theme identification, the authors reviewed the transcriptions independently.^33^ Regular meetings were conducted throughout the analysis to discuss and achieve consensus on identified codes and themes. Any ambiguities or interpretative discrepancies were meticulously examined and resolved through collaborative discussion. This iterative process strengthened the validity of our findings and ensured a comprehensive and nuanced interpretation of the data.

## Results

Theoretical descriptions were grouped into two themes based on similarities: “Development of metacognitive understanding” and “Cultivation of anxiety adjustment skills through metacognition.” Their corresponding subcategories are presented below, along with specific narratives: Representative nurses’ narratives are shown in italics, participants’ codes within [ ] after the quoted sections, and supplementary information in parentheses.

### Development of metacognitive understanding

Its three subthemes are listed below. First, recognition of the practical difficulties of metacognition through theoretical explanations. It was highlighted that for participants learning metacognition for the first time, to understand the concept of metacognition as “thinking about thinking” was not easy. They described their understanding upon receiving a theoretical explanation as follows:

“I have no idea what this is. It’s like, what is this?” [Participant A, Female, 56 years old, 35 years’ experience].

“I think it was about using knowledge to adjust your thinking, and change the way you perceive and look at things, but then, how do I actually use this knowledge?” [Participant B, Female, 37 years old, 15 years’ experience].

“Somehow, I thought it was still difficult to use this (metacognition) to think about some things. It was difficult to verbalize what metacognition is” [Participant E, Female, 31 years old, 9 years’ experience].

Second, development of practical understanding through scaffolding of metacognitive thought processes. The learning scaffold exercise helped the participants to develop a practical understanding of how to use metacognition to cope with anxiety. Their narratives are given below.

"I understand a little bit more, there. I thought it was like organizing what direction to take to reach a certain goal” [Participant A, Female, 56 years old, 35 years’ experience].

“In my abstract understanding of metacognition, it was like, ‘Okay; so, this is what it means,’ but through the exercise I learned that this is how nurses (who use metacognition) think, so my understanding has become more concrete, I think.” [Participant B, Female, 37 years old, 15 years’ experience].

“I found it difficult to capture the case studies with metacognition, which I still did not understand very well. However, I think, as I did the exercises, I gradually understood the way of thinking (metacognition).” [Participant E, Female, 31 years old, 9 years’ experience].

However, one participant said that his understanding of metacognition was insufficient, even after the scaffolding exercise.

“I was not yet sure about metacognition, when I did the exercise. I didn’t have a clear idea of how I should think about it” [Participant D, Female, 37 years old, 15 years’ experience].

Third, development of understanding by reflecting on practical anxiety experiences. The participants developed an understanding of what metacognition is about, by practicing it in their clinical practice after the workshop and verbalizing it in their online reflection sessions.

“I finally understood what it (anxiety adjustment through metacognition) means, after writing many reflections in my clinical practice.” [Participant D, Female, 37 years old, 15 years’ experience]

In particular, the teachers’ feedback about the students’ use of metacognition in the reflection sessions helped in deepening their understanding.

“I learned a lot from the teacher’s affirmation of what I had reflected upon, and the meaning the teacher gave to my reflections” [Participant B, Female, 37 years old, 15 years’ experience].

“When I received feedback that I was able to see things from a bird’s eye view, I thought, ‘Oh, yes, I am able to do that.’ It gave me confidence that my decisions and actions were not wrong. It was difficult (to look back with metacognition), but I appreciated the feedback” [Participant D, Female, 37 years old, 15 years’ experience].

### Cultivating anxiety adjustment skills through metacognition

As the participants’ understanding of metacognition progressed through the learning program, many of them talked about their awareness of anxiety, and what they had learned about coping with it. This category consisted of three subthemes, which are listed below.

First, cultivating awareness of focusing on anxiety. Participants acknowledged having become aware of their own state of anxiety, which they had previously not been aware of.

“I was unconsciously running away from anxiety. I was trying not to look at negative emotions because I didn’t want to see them, but (after the program) I started to look at anxiety from a bird’s eye view. I now try to be aware of the fact that I am feeling negative emotions” [Participant D, Female, 37 years old, 15 years’ experience].

“I originally had a tendency to be anxious. After hearing about metacognition, I started to think about my behavioral patterns” [Participant E, Female, 31 years old, 9 years’ experience].

Second, cultivating awareness of proactively coping with anxiety. The participants narratives are reproduced below.

“When I felt anxiety and wondered if it was appropriate to say such things or respond in such a way, I began to first become aware of the whole situation” [Participant A, Female, 56 years old, 35 years’ experience].

“I began to think about situations, in which, I tended to feel anxious. I also feel that my way of thinking has changed because now, when I feel anxious, instead of thinking that I am the only one who is in the wrong, I can resolve it by rethinking about the situation” [Participant B, Female, 37 years old, 15 years’ experience].

“Previously, I had a tendency to short-circuit my thinking (with regard to anxiety), and think in ambiguous ways. Now, I think, I have become more aware of what the problem is, and can look at the factors of anxiety in a comprehensive way” [Participant C, Male, 26 years old, 4 years’ experience].

Third, cultivating awareness of anxiety adjustment in patient care. The participants said using metacognition in actual patient care situations gave them a sense of being able to adjust their anxiety.

"Learning metacognition has helped me to think about what I can do in anxious situations. For example, I can now think of material for judgment, such as, if the patient is appealing to me now, it could be because there is trust between the patient and me, or the current appeal could be due to grief; so, listening is appropriate for now,” [Participant D, Female, 37 years old, 15 years’ experience].

“When I felt anxious, I used to think vaguely or worry about what to do, but by using metacognitive thinking, I was able to organize my anxiety and reduce it. It made it easier to cope” [Participant C, Male, 26 years old, 4 years’ experience].

## Discussion

This study investigated the efficacy of metacognitive approaches in nurses’ learning of anxiety adjustment. The results revealed that the program participants were able to apply metacognitive strategies to their own anxiety experiences, thereby improving their coping mechanisms. This chapter discusses two aspects of the impact of the metacognitive program on nurses and the potential contribution of metacognition to nurses’ anxiety adjustment training.

First, how the program promoted participants’ understanding of metacognition. Most participants (excluding Participant C) were learning about metacognition for the first time. Initially, many participants found the concept of “thinking about thinking,” one of the core elements of metacognition—abstract and difficult to understand. According to the cognitive load theory, the higher the complexity of a task, the lower the ability to process the task because cognitive resources are allocated to capturing the complexity.^34^^-^^37^Many participants found that the theoretical explanations of metacognition in the lecture did not help them understand the concept of “thinking about thinking.” Capturing both practical and metacognitive thinking may have imposed a high cognitive load on them and resulted in difficulties in understanding metacognitive thinking.

However, many participants were able to apply a metacognitive perspective to their own anxiety experiences through this program. In particular, the narratives of participants A, B, and E suggested that the program’s scaffolding exercises facilitated their understanding of metacognition. The challenge in teaching metacognition is said to be: How to induce metacognitive thinking in learners?^38^ In this program, using real situations that were easy for nurses to understand, and scaffolding to help them capture the metacognition inherent in the situations, may have bridged the gap between theory and practice in metacognition, and encouraged the induction of metacognitive thinking. Medina summarized that a useful teaching strategy for enhancing healthcare professionals’ metacognitive skills is to share goals with learners, and then ask them to think about what they know and do not know about the learning subject matter, and also about the difference between the two.^22^ In this program, we repeatedly explained to the participants that their goal was not to memorize theoretical knowledge of metacognition, but to think metacognitively about their own experiences. Hence, asking the participants during the sessions to think about the differences between the two cases—where metacognition worked and did not work—may have facilitated the learning of metacognition.

Second, the metacognitive approach’s potential for increasing resilience to cope with anxiety. Increasing resilience has been shown to reduce the negative effects of anxiety.^8^ It has been reported that a characteristic of individuals with high resilience is their ability to reflect on their anxiety,^2^^,^^16^ hence, educational approaches to enhance such reflective abilities are desirable.^15^ In this study, Participant C clearly stated that he was able to cope with anxiety more easily. Participant D also stated that she was able to think of ways to handle herself in situations of anxiety. These results suggest participation in the program led to an increase in resilience to anxiety.

The findings indicate that metacognition can effectively mitigate anxiety among nurses, thereby addressing a significant gap in current anxiety management programs. Metacognition has recently gained attention as an emerging method of anxiety regulation in psychiatry.^39^^-^^41^ For instance, Bailey and Wells' metacognitive program with 10 patients with health anxiety demonstrated that thought training to consciously recognize and adjust own anxiety improved anxiety levels over time.^40^ The application of metacognitive approaches to anxiety among healthcare professionals remains underexplored. Previous studies have focused on mindfulness-centered techniques addressing anxiety post-occurrence rather than during its onset. This study suggests that metacognition can effectively manage anxiety and boost confidence among healthcare professionals in hospital settings. Therefore, metacognitive training emerges as a promising new approach for anxiety regulation in healthcare professionals.

### Limitations

The study’s population included only Japanese nurses working at university hospitals in Japan, which may have led to biased learning from program participation. In addition, the small sample size made it difficult to demonstrate the program’s general effectiveness. A possible reason for the small sample size was that the concept of metacognition being new to the nurses. They may have found it difficult to relate the concept to their own nursing practice; hence, it might not have attracted much interest.

## Conclusions

Interviews and qualitative analysis revealed that participants moved from theoretical knowledge to practical application skills, thus demonstrating their understanding and application of metacognition for adjusting anxiety. Participants initially perceived metacognition as abstract and difficult to understand, but through the use of real-life examples of anxiety, scaffolding exercises to understand metacognitive concepts, and subsequent reflective practice, they developed a clearer and more actionable understanding of metacognition. It was suggested that this would facilitate positive efforts to adjust anxiety and improve coping in high-stress situations.

This study underscores the importance of incorporating metacognitive training into nursing education to enhance anxiety management. However, its limited sample size, which comprised a specific group of Japanese nurses, highlights the need for more extensive research to validate its findings in diverse healthcare settings.

Future studies should involve a broader and more diverse sample of nurses from diverse healthcare settings to investigate the efficacy of metacognitive training in anxiety management. Additionally, examining the multifaceted effects of learning metacognition, such as increased self-esteem and decreased anxiety levels, and determining the long-term impacts, may help to solidify the benefits of nurses learning metacognition.

### Acknowledgments

We thank the nurses who participated in this study. This study was supported by JSPS KAKENHI (Grant Number: JP 23K19820).

### Conflict of Interest

The author declares that there is no conflict of interest.

## Supplementary material

Supplementary file 1 Appendix A. Detail of the learning program

Supplementary file 2 Appendix B. Details of the participants

Supplementary file 3 Appendix C. Interview Guide

## References

[1] Feliciano AF, Feliciano EE, Al-Asiry S, Magtubo DJD, Reyes WS, Bautista EC, Santiango BMT, Gumabon RG (2022). Nurses’ stress, anxiety, depression, and burnout in the workplace: a correlational study.. International Journal of Advanced and Applied Sciences.

[2] Mirhendi H, Nishiyama Y, Rezaei-Matehkolaei A, Satoh K, Makimura K (2018). Erratum for Mirhendi et al., the first case of onychomycosis in a koala due to atypical isolates of , a diagnostic challenge.. Curr Med Mycol.

[3] Gao YQ, Pan BC, Sun W, Wu H, Wang JN, Wang L (2012). Anxiety symptoms among Chinese nurses and the associated factors: a cross sectional study.. BMC Psychiatry.

[4] Gray-Toft P, Anderson JG (1981). The nursing stress scale: development of an instrument.. Journal of Behavioral Assessment.

[5] Maharaj S, Lees T, Lal S (2018). Prevalence and risk factors of depression, anxiety, and stress in a cohort of Australian nurses.. Int J Environ Res Public Health.

[6] Patterson SL (2016). The effect of emotional freedom technique on stress and anxiety in nursing students: a pilot study.. Nurse Educ Today.

[7] Sajadi M, Goudarzi K, Khosravi S, Farahani MF, Mohammadbeig A. Benson’s relaxation effect in comparing to systematic desensitization on anxiety of female nurses: a randomized clinical trial. Indian J Med Paediatr Oncol. 2017;38(2):111-115. 10.4103/ijmpo.ijmpo_183_16 PMC5582545 28900316

[8] Banks J, Lopez V, Sahay A, Cleary M (2024). A scoping review of compassion fatigue among oncology nurses caring for adult patients.. Cancer Nurs.

[9] Maguire P (1985). Barriers to psychological care of the dying.. Br Med J (Clin Res Ed).

[10] Moore PM, Rivera S, Bravo-Soto GA, Olivares C, Lawrie TA (2018). Communication skills training for healthcare professionals working with people who have cancer.. Cochrane Database Syst Rev.

[11] National Cancer Center Japan. Cancer statistics in Japan 2023. 2023 [Cited 24 June 2024]; Available from: https://ganjoho.jp/public/qa_links/report/statistics/pdf/cancer_statistics_2023.pdf.

[12] Miyashita M, Onodera M, Kumata M, Ogiri N, Asano R, Ogasawara K, Goto A, Shibata H, Syoji Y, Sengoku M, Yamauti K, Monma N (2014). Difficulty with cancer care and related factors among nurses at Tohoku University Hospital.. Palliative Care Research.

[13] Koshino S, Aoyama M, Shoji Y, Saito A, Ogiri N, Hatakeyama R, Nakajo Y, Iinuma Y, Shida T, Monma N, Miyashita M (2019). Changes in difficulty with cancer care among nurses at Tohoku University hospital between 2010 and 2016.. Palliat Care Res.

[14] Kawanami A, Suzuki C. A literature review of perceptions of difficulty in decision support for cancer patients. Journal of Graduate School of Nursing Science Himeji University. 2022; 5:105-115 (In Japanese).

[15] Shikai N, Shono M, Kitamura T (2009). Effects of coping styles and stressful life events on depression and anxiety in Japanese nursing students: a longitudinal study.. Int J Nurs Pract.

[16] O'Rourke T, Budimir S, Pieh C, Probst T (2021). Psychometric qualities of the English Coping Scales of the Stress and Coping Inventory in a representative UK sample.. BMC Psychol.

[17] Minichiello V, Hayer S, Gillespie B, Goss M, Barrett B (2020). Developing a mindfulness skills-based training program for resident physicians.. Fam Med.

[18] Marconi A, Bàlzola MA, Gatto R, Soresini A, Mabilia D, Poletti S (2019). Compassion-oriented mindfulness-based program and health professionals: a single-centered pilot study on burnout.. European Journal of Mental Health.

[19] Krasner MS, Epstein RM, Beckman H, Suchman AL, Chapman B, Mooney CJ, Quill TE (2009). Association of an educational program in mindful communication with burnout, empathy, and attitudes among primary care physicians.. JAMA.

[20] Flavell JH (1979). Metacognition and cognitive monitoring: A new area of cognitive-developmental inquiry.. American Psychologist.

[21] Mohamed HM, Mohamed AI, Abdeen MA. The impact of metacognitive skills educational program on metacognitive awareness, self-efficacy, and problem solving skills among nursing students. American Journal of Nursing Research. 2020;8(2):289-296.

[22] Medina MS, Castleberry AN, Persky AM (2017). Strategies for Improving Learner Metacognition in Health Professional Education.. Am J Pharm Educ.

[23] Asadzandi S, Mojtahedzadeh R, Mohammadi A (2022). What are the Factors that Enhance Metacognitive Skills in Nursing Students? A Systematic Review.. Iran J Nurs Midwifery Res.

[24] Yousefi M, Barzegar M, Kouroshnia M, Khayyer M (2021). Investigating the mediating role of cognitive emotion regulation strategies in the relationship between meta-cognitive beliefs and learning anxiety.. Iranian Evolutionary and Educational Psychology.

[25] Iwama Y, Ikeda M. A metacognitive skill education method for outpatient cancer nurse to alleviate their anxiety. JSiSE Research Report. 2020;35(1):19-26. (In Japanese).

[26] Patton MQ. Qualitative research & evaluation methods. 4th edition. Saint Paul, MN: SAGE Publications, Inc; 2014.

[27] Otani T. “SCAT” a qualitative data analysis method by four-step coding: easy startable and small scale data-applicable process of theorization. Bulletin of the Graduate School of Education and Human Development Education Sciences. 2008;54(2):27-44 (In Japanese).

[28] Otani T. Paradigm and design of qualitative study: from research methodology to SCAT. Nagoya: The University of Nagoya Press; 2019. (In Japanese).

[29] Soemantri D, Greviana N, Findyartini A, Azzahra TB, Suryoadji KA, Mustika R, Felaza E (2021). "To obey or not to obey" - Medical students' response towards professional dilemmas in a hierarchical and collectivist culture.. PLoS One.

[30] Suematsu M, Okumura K, Hida T, Takahashi N, Okazaki K, Fuchita E, Abe K, Kamei H, Hanya M (2021). Students' perception of a hybrid interprofessional education course in a clinical diabetes setting: a qualitative study.. Int J Med Educ.

[31] Okazaki K, Takahashi N, Shingaki T, Perez-Nieves M, Stuckey H (2022). Key factors for overcoming psychological insulin resistance: A qualitative study in Japanese people with type 2 diabetes.. Prim Care Diabetes.

[32] Seki M, Fujinuma Y, Matsushima M, Joki T, Okonogi H, Miura Y, Ohno I (2019). How a problem-based learning approach could help Japanese primary care physicians: a qualitative study.. Int J Med Educ.

[33] Stahl NA, King JR. Understanding and using trustworthiness in qualitative research. Journal of Developmental Education. 2020;44(1):26-28.

[34] Pieschl S, Stahl E, Murray T, Bromme R (2012). Is adaptation to task complexity really beneficial for performance?. Learning and Instruction.

[35] Rogers BA, Franklin AE (2021). Cognitive load experienced by nurses in simulation-based learning experiences: an integrative review.. Nurse Educ Today.

[36] van Merriënboer JJ, Sweller J (2010). Cognitive load theory in health professional education: design principles and strategies.. Med Educ.

[37] Sweller J (1988). Cognitive load during problem solving: effects on learning.. Cognitive Science.

[38] Sawyer RK, The Cambridge handbook of the learning sciences. 2nd edition. Cambridge: Cambridge University Press; 2014.

[39] Melli G, Carraresi C, Poli A, Bailey R (2016). The role of metacognitive beliefs in health anxiety.. Personality and Individual Differences.

[40] Bailey R, Wells A (2024). Feasibility and preliminary efficacy of metacognitive therapy for health anxiety: a pilot RCT.. Journal of Affective Disorders Reports.

[41] Keen E, Kangas M, Gilchrist PT (2022). A systematic review evaluating metacognitive beliefs in health anxiety and somatic distress.. Br J Health Psychol.

