# Supplementary material for: Exploring a metacognitive approach for case analysis based learning of anxiety adjustment in nurses: a qualitative study
Source: Int J Med Educ. 2024 Aug 23;15:99–109. doi: 10.5116/ijme.66af.82fc (PMC11687377; doi:10.5116/ijme.66af.82fc)
Supplement: Supplementary file 1 — Appendix A. Detail of the learning program [file ijme-15-99-S1.pdf]

Appendix A

Details of the learning program

Sessions	Duration	Materials	Activities	Learning Goals
Classroom Workshop (In-person, small group)				
1. Lecture<br>Introduction to metacognition and how anxiety is analyzed	30 min	・Lecture material comprising 40 slides.<br>(Contents：Basic metacognitive theory and detailed guidance on case analysis)	・Share personal experiences of anxiety, guided by the lecturer to bring attention to clinical anxiety.<br>・Engage in lectures on metacognition, where the lecturer explains basic knowledge of the metacognitive theory and the benefits of metacognition in adjusting anxiety.	・Look back at personal clinical anxieties.<br>・Have a basic knowledge of metacognitive theory.
2. Case Analysis<br>Analysis of cases where metacognition was applied versus not applied in anxiety situations	30 min	・The metacognitive analysis model of nurses' anxiety adjustment.<br>・Two scenarios illustrating nurses' thoughts and behaviors in anxiety-inducing situations: one with effective metacognitive anxiety regulation and one without.<br>・Exemplars of the two-scenario analysis.	・Analyze two cases in which metacognition was either applied or not applied in anxiety adjustment, utilizing the analytical model.<br>・Compare the model answers in the analysis with the participant's own answers<br>・Evaluate the differences in outcomes based on the presence or absence of metacognition.	・Find out the role of metacognition in adapting to anxiety in clinical settings.<br>・Explore strategies for applying metacognition to manage anxiety.
3. Reflection:<br>Reflection on personal anxiety experiences using metacognitive analysis model	30 min	・Analysis model (same as used in case analysis)<br>・A sheet for participants to document their analysis of their own anxiety.	・Reflect on and verbalize personal anxiety experiences.<br>・Receive feedback from the instructor on metacognitive perspectives.	・Facilitate metacognitive self-awareness regarding anxiety experiences.
Online Reflection (Google Form, Individual)				
Session 1 (Immediate):<br>Reflection on anxiety experiences	15-30 min	・Questions for introspection about anxiety<br>(Contents: Self-awareness of anxiety, Metacognitive perception of anxiety, Awareness of changes in participant's thinking about anxiety, Goal setting for anxiety management)<br>・Analysis model (same as used in case analysis)	・Metacognitive reflection on participant's own anxiety with the analysis model.<br>・Try to articulate participants thoughts about anxiety.<br>・Receive feedback from the instructor about the representation.	・Become aware of the concept of metacognitive reflection.
Session 2 (Two weeks later)<br>Reflection on anxiety experiences	15-30 min	Questions for introspection about anxiety<br>(Contents: Same as session 1)	・Metacognitive reflection on participant's own anxiety by themself.<br>・Try to articulate participants thoughts about anxiety.<br>・Receive feedback from the instructor about the representation.	・Start considering the metacognition of participant's own anxiety without relying on an analytical model of metacognition.
Session 3 (One month later)<br>Reflection on anxiety experiences	15-30 min	Questions for introspection about anxiety<br>(Contents: Same as session 1)	・Metacognitive reflection on participant's own anxiety by themself.<br>・Try to articulate participants thoughts about anxiety.<br>・Receive feedback from the instructor about the representation.	・Enhance the perception of anxiety adjustment via metacognition.

Session 4 (Two months later)<br>Reflection on anxiety experiences	15-30 min	Questions for introspection about anxiety<br>(Contents: Same as session 1)	・Metacognitive reflection on participant's own anxiety by themself.<br>・Try to articulate participants thoughts about anxiety.<br>・Receive feedback from the instructor about the representation.	・Enhance the perception of anxiety adjustment via metacognition.
Session 5 (Three months later)<br>Reflection on anxiety experiences	15-30 min	Questions for introspection about anxiety<br>(Contents: Same as session 1)	・Metacognitive reflection on participant's own anxiety by themself.<br>・Try to articulate participants thoughts about anxiety.<br>・Receive feedback from the instructor about the representation.	・Enhance the perception of anxiety adjustment via metacognition.
