# Supplementary material for: Exploring a metacognitive approach for case analysis based learning of anxiety adjustment in nurses: a qualitative study
Source: Int J Med Educ. 2024 Aug 23;15:99–109. doi: 10.5116/ijme.66af.82fc (PMC11687377; doi:10.5116/ijme.66af.82fc)
Supplement: Supplementary file 2 — Appendix B. Details of the participants [file ijme-15-99-S2.pdf]

Appendix B

Details of the participants

Participant codes	Age	Sex	Years experience	Current department	Academic background
A	56	Female	35	Outpatient Unit (Internal Medicine)	Associate Degree
B	37	Female	15	Outpatient Unit (Internal Medicine)	Master
C	26	Male	4	Inpatient Unit (Urology)	Bachelor
D	37	Female	15	Outpatient Unit (Internal Medicine)	Bachelor
E	31	Female	9	Inpatient Unit (Intensive Care Unit)	Bachelor
