# Supplementary material for: Exploring a metacognitive approach for case analysis based learning of anxiety adjustment in nurses: a qualitative study
Source: Int J Med Educ. 2024 Aug 23;15:99–109. doi: 10.5116/ijme.66af.82fc (PMC11687377; doi:10.5116/ijme.66af.82fc)
Supplement: Supplementary file 3 — Appendix C. Interview Guide [file ijme-15-99-S3.pdf]

Appendix C

Interview Guide

1. How has your attitude towards anxiety evolved after participating in the program compared to your attitude prior to the program?
2. When you were introduced to the concept of metacognition during the lecture, what were your initial thoughts and understanding of it?
3. How has your understanding of metacognition changed through the case analysis?
4. How has your understanding of metacognition been influenced by analyzing your experiences of anxiety from a metacognitive perspective?
5. How has your understanding of metacognition been shaped by the feedback provided by your instructor on your online metacognitive reflections?
